# AI-Based Ultrasound Nomogram for Differentiating Invasive from Non-Invasive Breast Cancer Masses

**DOI:** 10.3390/cancers17152497

**Published:** 2025-07-29

**Authors:** Meng-Yuan Tsai, Zi-Han Yu, Chen-Pin Chou

**Affiliations:** 1 Department of Radiology, Kaohsiung Veterans General Hospital, Kaohsiung 813414, Taiwan; mytsai@vghks.gov.tw (M.-Y.T.); wanjhenyu@gmail.com (Z.-H.Y.); 2 Department of Pharmacy, College of Pharmacy, Tajen University, Pingtung 900391, Taiwan; 3 Department of Medical Imaging and Radiology, Shu-Zen Junior College of Medicine and Management, Kaohsiung 813414, Taiwan; 4 School of Medicine, National Yang Ming Chiao Tung University, Taipei 112304, Taiwan; 5 Department of Radiology, Jiannren Hospital, Kaohsiung 813414, Taiwan; 6 Department of Medical Laboratory Science and Biotechnology, Fooyin University, Kaohsiung 813414, Taiwan

**Keywords:** breast cancer, ductal carcinoma in situ, ultrasound, nomogram, logistic regression, artificial intelligence, S-detect system

## Abstract

**Purpose:** This study aimed to develop a predictive nomogram integrating AI-based BI-RADS lexicons and lesion-to-nipple distance (LND) ultrasound features to differentiate mass-type ductal carcinoma in situ (DCIS) from invasive ductal carcinoma (IDC) visible on ultrasound. **Methods:** The final study cohort consisted of 170 women with 175 pathologically confirmed malignant breast lesions, including 26 cases of DCIS and 149 cases of IDC. LND and AI-based features from the S-Detect system (BI-RADS lexicons) were analyzed. Rare features were consolidated into broader categories to enhance model stability. Data were split into training (70%) and validation (30%) sets. Logistic regression identified key predictors for an LND nomogram. Model performance was evaluated using receiver operating characteristic (ROC) curves, 1000 bootstrap resamples, and calibration curves to assess discrimination and calibration. **Results:** Multivariate logistic regression identified smaller lesion size, irregular shape, LND ≤ 3 cm, and non-hypoechoic echogenicity as independent predictors of DCIS. These variables were integrated into the LND nomogram, which demonstrated strong discriminative performance (AUC = 0.851 training; AUC = 0.842 validation). Calibration was excellent, with non-significant Hosmer-Lemeshow tests (*p* = 0.127 training, *p* = 0.972 validation) and low mean absolute errors (MAE = 0.016 and 0.034, respectively), supporting the model’s accuracy and reliability. **Conclusions:** The AI-based comprehensive nomogram demonstrates strong reliability in distinguishing mass-type DCIS from IDC, offering a practical tool to enhance non-invasive breast cancer diagnosis and inform preoperative planning.

## Simple Summary

This study developed a predictive nomogram that integrates AI-extracted ultrasound features—derived from the ACR BI-RADS lexicon—and lesion-to-nipple distance (LND) to differentiate mass-type ductal carcinoma in situ (DCIS) from invasive ductal carcinoma (IDC). Key ultrasound features, including lesion size, shape, echogenicity, and LND, were identified as independent predictors. The resulting nomogram demonstrated robust discriminative performance and excellent calibration, underscoring its potential as a reliable, non-invasive tool for differentiating mass-type DCIS from IDC.

## 1. Introduction

Breast cancer ranked as the most frequently diagnosed malignancy worldwide in 2020, with over 2.3 million new cases and approximately 0.7 million cancer-related deaths [1]. Ductal carcinoma in situ (DCIS), a non-invasive cancer confined to the mammary ducts and ductal-lobular system, is considered a potential precursor of invasive ductal carcinoma (IDC) [2] with a 20% to 53% risk of progressing to IDC [3]. Accurate differentiation of DCIS from IDC is crucial for appropriate management before surgical intervention; however, differentiating DCIS from IDC remains challenging due to overlapping imaging features and technical limitations [4].

In the detection of breast cancer in dense breast tissue, ultrasound is superior to mammography due to its higher sensitivity and specificity [5]. IDC often presents as irregular hypoechoic masses, while microcalcifications in DCIS typically appear as echogenic foci, and detection may be difficult due to the subtle nature of these findings [6]. The highly variable ultrasound appearance of DCIS, ranging from mass-type lesions to non-mass lesions, often overlaps with imaging characteristics of IDC, making differentiation challenging [7]. Moreover, the diagnostic accuracy of ultrasound in distinguishing IDC from DCIS heavily depends on the radiologist’s experience, which may lead to subjectivity and affect diagnostic reliability [8]. B-mode ultrasound shows variable diagnostic performance for mass-like and non-mass-like breast lesions, with accuracy enhanced by combining contrast-enhanced ultrasound and shear wave elastography [9].

To overcome limitations of conventional diagnostic ultrasound, standardized BI-RADS features have been utilized as radiomics elements to improve the differentiation of mass-type DCIS from IDC beyond visual assessment [10]. Recent studies have further shown that radiomics models based on BI-RADS descriptors can effectively differentiate DCIS from IDC, potentially enhancing diagnostic precision and aiding treatment planning [11]. Advancements in AI have improved diagnostic reliability by standardizing image acquisition, automating parameter adjustments, providing real-time quality feedback, and enabling consistent interpretation of several imaging features for more accurate lesion characterization [12,13,14,15].

The S-Detect system exemplifies these technological advances, with validation studies consistently demonstrating high diagnostic accuracy in distinguishing benign from malignant breast lesions [16]. Systematic characterization of ultrasound features in non-invasive breast cancer by imaging specialists has further identified key diagnostic attributes that enhance both accuracy and reliability [5]. While recent studies have explored advanced methodologies including optical coherence tomography [17], multi-parametric biomarkers [18], ultrasound-based radiomic prediction tool [19], and multimodal imaging features [20,21] with excellent research performance, their high cost and technical complexity limit clinical adoption, emphasizing the need for accessible AI-enhanced tools. We represent a novel paradigm by integrating simple anatomical measurements, specifically lesion-to-nipple distance (LND), with AI-standardized imaging features to create a clinically accessible diagnostic tool. This methodology circumvents the need for complex radiomics software or extensive feature extraction protocols, thereby facilitating broader clinical implementation.

## 2. Materials and Methods

### 2.1. Study Population

This retrospective study, conducted at Kaohsiung Veterans General Hospital, was approved by the Institutional Review Board, which waived the requirement for informed consent due to the retrospective nature of the study.

**Figure 1:**
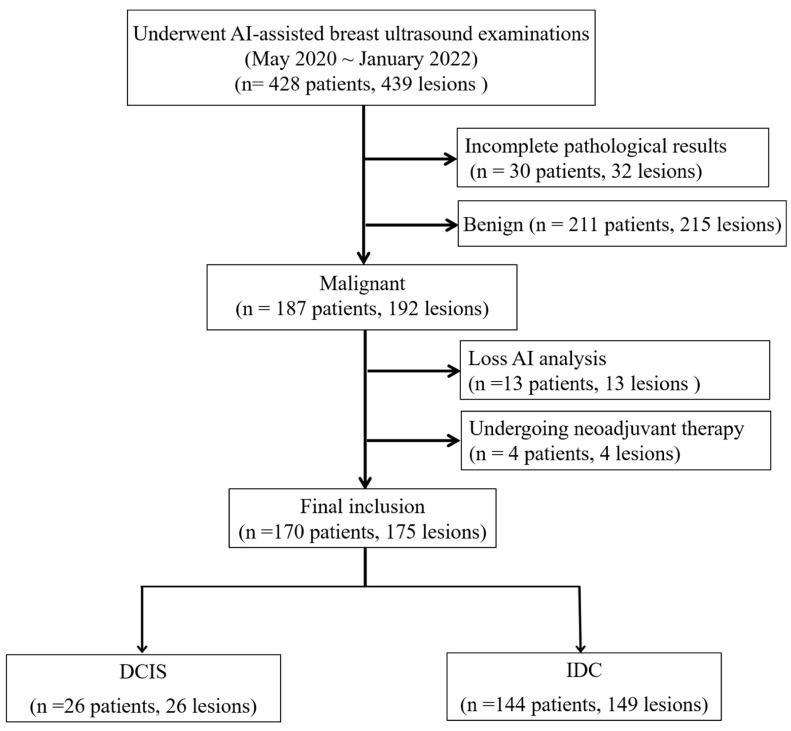
Flowchart depicting patient enrollment and case selection process.

Between May 2020 and January 2022, 428 women underwent breast ultrasound examinations using the S-Detect AI system (Samsung Medison Co., Seoul, Republic of Korea). Following surgical intervention, pathological assessment confirmed 192 malignant breast lesions. In this retrospective study, we analyzed the pre-surgical ultrasound images and their corresponding S-Detect outputs in relation to the final pathological diagnoses. We excluded lesions with inadequate imaging for comprehensive AI analysis (*n* = 13), including insufficient image quality, incomplete lesion visualization, technical errors, or non-mass lesions that could not be adequately characterized using BI-RADS mass descriptors and patients who had received neoadjuvant therapy prior to imaging (*n* = 4). The final cohort included 170 women with 175 malignant breast lesions, comprising 149 IDC and 26 DCIS, including 4 patients with two IDC lesions and 1 patient with both IDC and DCIS (Figure 1).

### 2.2. Image Acquisition

Breast ultrasound examinations were performed by one of four experienced sonographers, each possessing 10–25 years of expertise in breast imaging. The examinations were performed using the Samsung RS85 ultrasound system equipped with a 3–12 MHz (L3-12A) linear transducer. Patients were positioned supine with the ipsilateral arm raised to optimize breast exposure during the examination. Real-time imaging captured longitudinal and transverse views of suspicious lesions, with a focus on static ultrasound images that highlighted the lesion’s longest diameter.

To mitigate automation bias, experienced sonographers applied independent clinical judgment during image acquisition despite AI (S-Detect) suggestions, following standardized protocols after formal system training. All ultrasound images were independently reviewed by a board-certified breast radiologist, blinded to both AI outputs and pathological findings, ensuring objective assessment. Regular consensus meetings helped minimize inter-observer variability. The nomogram served solely as a clinical decision-support tool, requiring manual data input and interpretation to maintain physician-driven diagnostic decisions.

Each lesion was assigned a unique identifier for precise tracking, and key images were analyzed using the S-Detect system. Sonographers outlined lesion interiors using a graphical interface and selected the optimal boundary contour suggested by the S-Detect system. The S-Detect system (Samsung Medison, Seoul, Republic of Korea) is an FDA-cleared AI-powered ultrasound platform utilizing a GoogLeNet-based CNN trained on 7408 breast images from 5151 patients (57.4% benign, 42.6% malignant) that operates through three sequential steps: lesion boundary segmentation, BI-RADS lexicon feature extraction, and benign/malignant classification [22]. BI-RADS lexicon extraction utilizes a multi-branch AlexNet architecture that processes three preprocessed margin-variant images through convolutional layers. Channels extracted features through fully connected layers with soft max activation to output five standardized BI-RADS descriptors (shape, orientation, margin, posterior features, and echo pattern) with probabilistic confidence for clinical decision support [22].

### 2.3. AI Feature Extraction and Data Collection

The lesion-to-nipple distance (LND), defined as the shortest distance from the lesion to the nipple, and the lesion depth (in cm) from the skin surface were recorded to determine lesion location. Additional imaging features, such as lesion area (calculated in cm^2^ based on the AI-defined region of interest, ROI), were analyzed.

**Figure 2:**
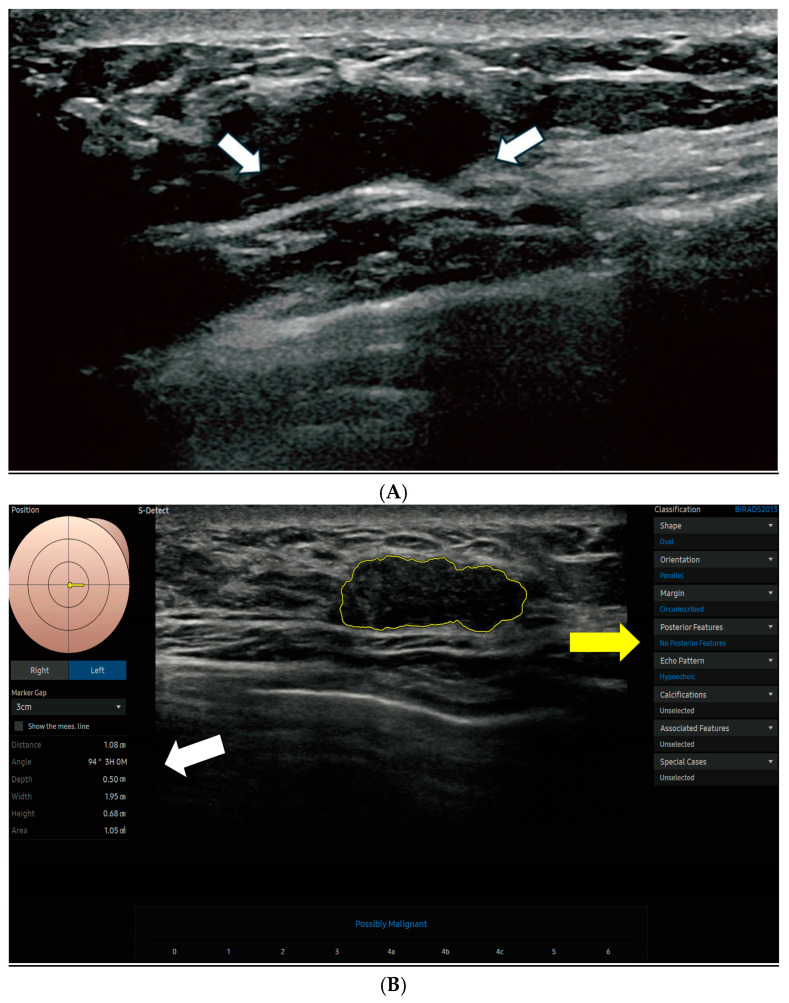
Breast ultrasound and S-Detect AI analysis in a 44-year-old woman with a BI-RADS 4A lesion. (**A**) B-mode ultrasound image of the left breast demonstrates a hypoechoic lesion (arrows), initially categorized as BI-RADS 4A by the interpreting radiologist. (**B**) The S-Detect AI delineates the lesion margins and provides associated quantitative size parameters (white arrow): distance, 1.08 cm; angle, 94°; depth, 0.50 cm; width, 1.95 cm; height, 0.68 cm; and area, 1.05 cm^2^. The AI-generated BI-RADS lexicon assessment (yellow arrow) characterizes the lesion as oval, parallel-oriented, circumscribed, hypoechoic, and without posterior acoustic features.

The S-Detect AI system accurately outlined lesion boundaries and calculated the ROI area, which helps standardize analytical measurements across cases. Morphological characteristics were evaluated based on the 5th edition of the ACR BI-RADS lexicon, including shape (oval, round, lobular, or irregular), orientation (parallel or non-parallel), margin (circumscribed, indistinct, angular, microlobulated, or spiculated), posterior acoustic features (none, enhancement, shadowing, or combined), and internal echo patterns (anechoic, hyperechoic, isoechoic, hypoechoic, or complex). Figure 2 displays the S-Detect AI system’s processed ultrasound images of lesions.

### 2.4. AI-Driven Model Development and Validation

The dataset was randomly partitioned into training (70%) and validation (30%) sets using the R function ‘createDataPartition,’ aiming to achieve a uniform distribution of key variables across both sets. This technique can establish uniform representation of crucial variables in both sets, resulting in a more robust model with reduced bias and enhanced predictive accuracy and generalizability [23,24]. The training set was used to assess variables and develop the model, while the validation set was employed to verify the outcomes generated by the training set.

Univariate and multivariable logistic regression analyses were used to develop a DCIS predictive model. To minimize estimate instability in logistic regression, we grouped subcategories based on preliminary univariate analysis that showed no statistically significant differences (*p* > 0.20) and overlapping odds ratios, indicating similar diagnostic impact and comparable behavior. Variables with *p* < 0.1 in the univariate analysis were included in the multivariable analysis, where backward elimination was performed to identify independent risk factors, sequentially removing non-significant variables. These thresholds (*p* > 0.20 and *p* < 0.1) were selected based on clinical prediction modeling literature to optimize model stability and retain potentially informative variables in a small-sample setting [25]. A threshold of *p* < 0.1 was used for both variable selection and elimination to prevent potential predictors from not being prematurely excluded. Based on these predictors, the ‘LND nomogram’ model was developed to estimate the probability of DCIS. Additionally, the ‘AI nomogram’ model was created, incorporating key AI-derived ultrasound features, to assess the added value of AI in DCIS risk prediction.

A nomogram was developed using a multivariable logistic regression model to predict DCIS in malignant lesions. Internal validation was performed using two approaches: (1) bootstrap resampling with 1000 iterations and (2) testing on an internal held-out validation set (30% of data) from the same institution. Note that external validation with data from other institutions was not performed in this proof-of-concept study. Discrimination was evaluated using the area under the receiver operating characteristic curve (AUC), which measures the model’s ability to distinguish between DCIS and IDC cases. Higher AUC values (closer to 1) indicated superior discriminative accuracy [26]. The calibration curves demonstrated strong agreement between predicted and observed outcomes, confirming the model’s reliability for clinical probability estimation, while the high AUC values confirmed excellent discriminative performance [27]. Calibration was assessed using mean absolute error (MAE) and calibration curves with the Hosmer-Lemeshow test, measuring the agreement between predicted probabilities and observed outcomes. Lower MAE values (closer to 0) indicated better calibration, meaning the predicted probabilities more accurately reflected actual risk [28,29]. In addition, accounting for the inherent class imbalance between DCIS and IDC cases in our dataset, Matthews Correlation Coefficient (MCC) and Cohen’s Kappa were specifically employed to assess classification agreement.

### 2.5. Statistical Analysis

R software (version 4.2.3 and R Studio version 2022.07.1) and SPSS 25.0 were used to perform the statistical analysis. Continuous variables were expressed as means ± standard deviation (SD). The *t*-test was used for comparing normally distributed continuous variables, while the chi-square test was applied to analyze categorical variables. The Hosmer-Lemeshow test yielded a *p* > 0.05, indicating good model calibration and accurate prediction of outcomes across its range. The rms package in R was used to construct and evaluate the nomograms, while the pROC package was applied to generate ROC curves and calculate AUC values. All statistical tests were two-sided, with a significance threshold of *p* < 0.05.

## 3. Results

### 3.1. Patient Demographics

A total of 170 patients (overall mean age = 58.2 years) with 175 pathologically confirmed malignant breast lesions were included in the study. Clustering bias was considered negligible in that only 2.9% of patients had multiple lesions. The mean patient age was 53.2 years (SD = 13.5) in the training set and 54.7 years (SD = 12.8) in the validation set. Patients were categorized into two age groups: ≤50 years and >50 years. In the training set, 28.7% of patients were ≤50 years, while 71.3% were >50 years. The validation set exhibited a similar distribution, with 26.4% and 73.6% in the respective age groups. No significant difference in age distribution was observed between the two sets (*p* = 0.900), supporting demographic balance and helping to reduce potential selection bias (Table 1).

### 3.2. Lesion and Ultrasound Characteristics

Each breast lesion was analyzed independently. The mean pathological sizes were 1.35 cm (SD = 0.42) for DCIS and 2.10 cm (SD = 0.87) for IDC. As shown in Table 1, the study included 122 lesions in the training set and 53 in the validation set. The proportions of DCIS were 15.6% in the training set and 13.2% in the validation set, while IDC accounted for 84.4% and 86.8%, respectively. No significant difference was observed (*p* = 0.863), supporting consistency in lesion characteristics across groups.

Key predictive variables exhibited similar distributions between the training and validation sets. LND was comparable (LND ≤ 3 cm: 62.3% vs. 58.5%; LND > 3 cm: 37.7% vs. 41.5%, *p* = 0.760), as was mean lesion area (2.33 ± 2.38 cm^2^ vs. 2.32 ± 2.07 cm^2^, *p* = 0.416). Irregular shape was the most common morphology (75.4% vs. 81.1%), with no significant differences across other shapes (oval: 13.1% vs. 9.4%; round: 7.4% vs. 7.5%; lobular: 4.1% vs. 1.9%, *p* = 0.776). Echo patterns were also similar (*p* = 0.778), with hypoechoic lesions being the most frequent (81.1% vs. 84.9%), followed by isoechoic (14.8% vs. 9.4%), complex (2.5% vs. 3.8%), and hyperechoic (1.6% vs. 1.9%) patterns. The overall consistency between the training and validation sets supports the reliability of subsequent predictive modeling.

### 3.3. Variable Analysis and Model Development

Based on preliminary univariate analysis, subcategories such as oval, round, and lobular shapes (OR: 0.12–0.18), and angular and spiculated margins (OR: 0.65–0.71), were grouped due to overlapping odds ratios and non-significant differences (*p* > 0.20), indicating comparable diagnostic behavior. The final AI-derived dataset included the following variables: lesion area (cm^2^), depth (cm), shape (irregular, oval/round/lobular), orientation (non-parallel, parallel), margin (microlobulated, indistinct, circumscribed, angular/spiculated), posterior features (shadowing, non-shadowing), and echo pattern (hypoechoic, non-hypoechoic).

Table 2 summarizes the logistic regression analysis results for the training set. Variables with *p* < 0.1 in the univariable analysis were included in the multivariable logistic regression, while non-significant variables such as depth, margin types, posterior features, and age (*p* ≥ 0.1) were excluded to refine the predictive model. Among the significant predictors (*p* < 0.1) for DCIS probability estimation, lesion area, LND, lesion shape, and echoic pattern were identified as key factors. A shorter LND (≤3 cm) was strongly associated with a higher risk of DCIS (OR = 9.244, 95% CI: 1.778–48.087, *p* = 0.011), suggesting an inverse relationship between LND and DCIS likelihood. Similarly, smaller lesion areas were linked to a higher likelihood of DCIS (OR = 0.213, 95% CI: 0.066–0.686, *p* = 0.012), further supporting the inverse relationship between lesion size and DCIS likelihood.

In terms of morphology, irregular lesion shape was significantly more associated with DCIS than oval, round, or lobular lesions (OR = 9.736, 95% CI: 1.088–87.965, *p* = 0.042). Additionally, non-hypoechoic patterns were more strongly linked to DCIS risk compared to hypoechoic lesions (OR = 3.427, 95% CI: 0.984–11.934, *p* = 0.066).

**Figure 3:**
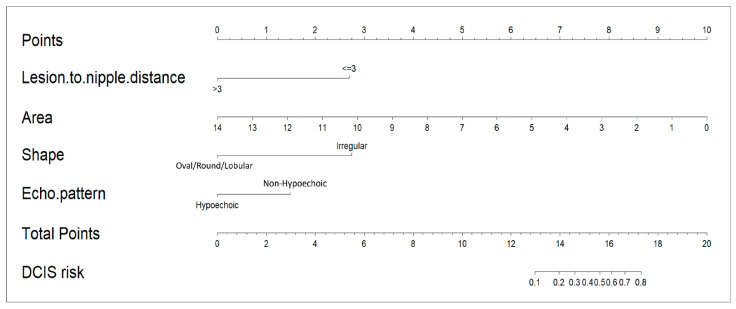
Nomogram for predicting mass-type non-invasive breast cancer based on ultrasound images. The top row shows the point assignment for each variable. Rows 2–5 list the individual predictor variables in the nomogram, with point values based on mass characteristics. The total points are summed in row 6, and the bottom row indicates the probability of non-invasive breast cancer.

The LND nomogram (Figure 3) combined LND, lesion area, lesion shape, and echoic pattern, incorporating both anatomical and morphological factors for prediction. In contrast, the AI nomogram utilized only AI-extracted ultrasound features (shape and echo pattern). We developed these two distinct models to evaluate the added value of integrating clinical measurements with AI-derived features. This distinction highlights the LND nomogram’s more comprehensive predictive model, while the AI nomogram remains limited to feature-based analysis.

### 3.4. Nomogram Validation and Performance Assessment

**Figure 4:**
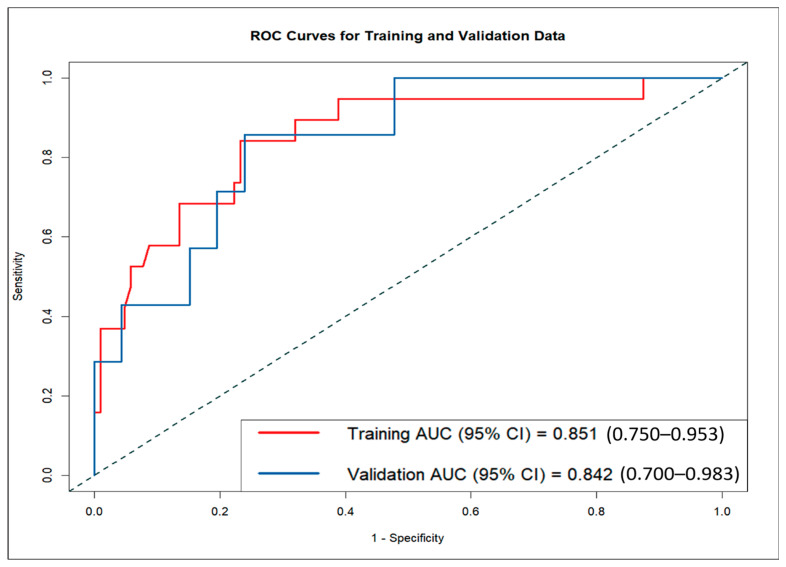
ROC curves showing comparative diagnostic performance of the nomogram in the training set (red) and validation set (blue).

The LND nomogram demonstrated strong discriminatory ability based on ROC curve analysis, with AUCs of 0.851 (95% CI: 0.810–0.892) for the training set and 0.842 (95% CI: 0.774–0.910) for the validation set (Figure 4). In contrast, the AI nomogram, based solely on AI-derived features, yielded AUCs of 0.691 for the training set and 0.667 for the validation set. These findings underscore the superior predictive accuracy of the LND nomogram over the AI nomogram in both training and validation sets, demonstrating the significant improvement achieved by combining clinical measurements with AI-extracted features.

**Figure 5:**
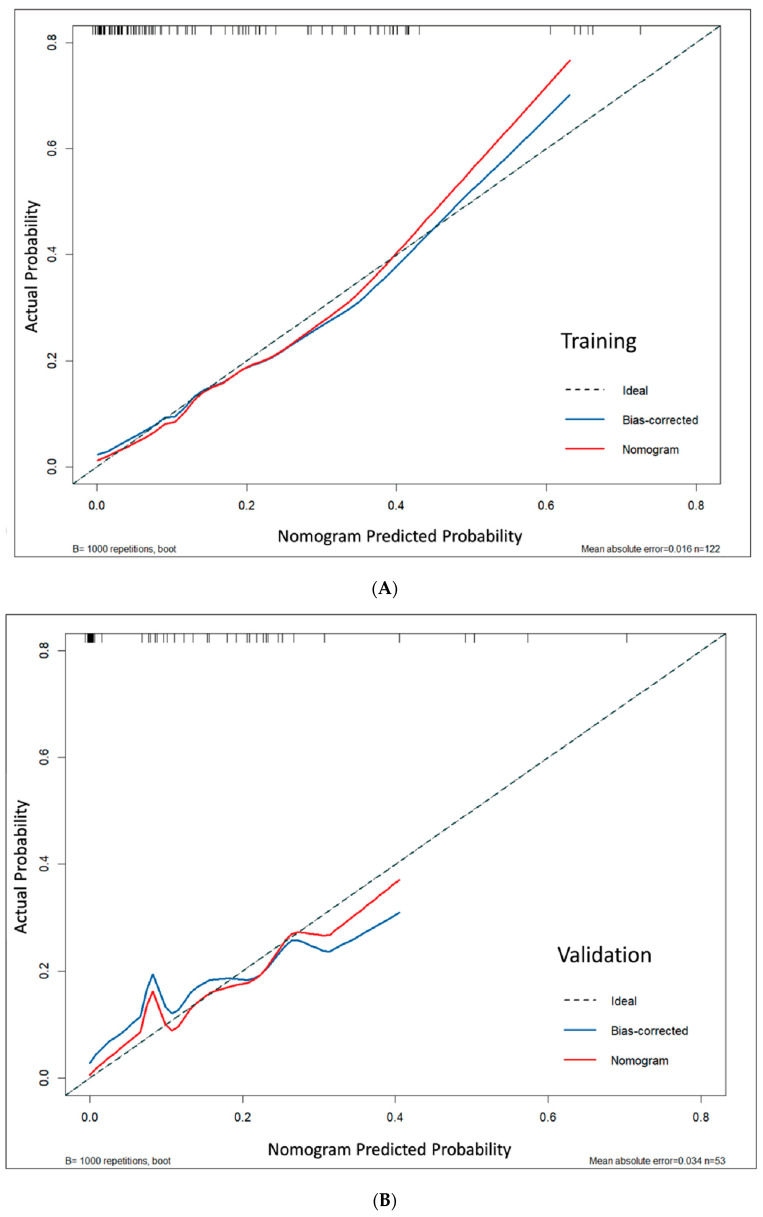
Calibration plots showing nomogram predictions versus actual observations in (**A**) the training set and (**B**) the validation set. The proximity of the calibration curves to the diagonal reference line indicates excellent model performance.

Model calibration demonstrated excellent agreement between predicted and actual probabilities. This was supported by non-significant Hosmer–Lemeshow test results (*p* = 0.127 for the training set and 0.972 for the validation set; both *p* > 0.05), low mean absolute errors (MAE = 0.016 and 0.034), and calibration curves that closely followed the ideal diagonal line, indicating reliable risk prediction across the full probability spectrum (Figure 5). Bootstrap analysis confirmed consistent calibration performance across probability ranges (Figure S1).

Diagnostic metrics showed consistent performance across cohorts, with sensitivity/specificity of 84.2%/87.4% (training) and 85.7%/87.0% (validation). The false negative rates were low (15.8% training, 14.3% validation), and false positive rates were moderate (12.6% training, 13.0% validation), indicating reliable DCIS detection with acceptable specificity. MCC values were 0.639 (training) and 0.594 (validation), and Cohen’s Kappa values were 0.634 and 0.586, respectively. These indicate moderate to substantial agreement despite class imbalance, supporting the nomogram’s reliability in distinguishing mass-type DCIS from IDC.

## 4. Discussion

This study analyzed 175 malignant breast lesions from 170 female patients to develop a comprehensive nomogram for differentiating DCIS from IDC, incorporating both traditional ultrasound features and AI-based imaging techniques. The LND nomogram incorporated variables such as LND and AI-analyzed lesion characteristics, including area, shape, and echo patterns. The nomogram achieved good discriminative performance with AUCs of 0.851 in the training set and 0.842 in the validation set, suggesting promising potential for clinical application, though external validation with larger multicenter cohorts is essential to confirm broader clinical utility. Calibration curves confirmed a high degree of alignment between predicted and observed outcomes in both the training and validation sets, further validating the model’s accuracy. We developed and validated an AI-based ultrasound nomogram using ACR BI-RADS features to differentiate mass-type invasive from non-invasive breast cancers, potentially transforming preoperative treatment planning by preventing DCIS overtreatment while ensuring appropriate IDC management.

Several ultrasound-based studies have explored methods to predict DCIS and differentiate it from IDC, utilizing diagnostic tools such as B-mode, color Doppler, elastography, and contrast-enhanced ultrasound (CEUS) [26,29,30]. Marco Moschetta et al. found that DCIS frequently appears as mass-like hypoechoic lesions with indistinct margins on B-mode ultrasound [30]. Another study highlighted that microinvasive DCIS exhibits increased vascularization and mixed vascular distribution on color Doppler sonography compared to pure DCIS [28]. Shi et al. demonstrated that elastography identified significantly higher edge shear wave velocity (SWV) in IDC compared to DCIS, achieving moderate diagnostic accuracy comparable to diffusion-weighted MRI [29]. CEUS exhibited diagnostic accuracy comparable to conventional ultrasound, excelling in distinguishing DCIS from invasive cancers with a 94% true-positive rate based on shape, margin clarity, and enhancement patterns [26]. Our study developed and validated a predictive model for enhancing DCIS diagnosis, integrating AI-based breast ultrasound and LND. These findings align with previous studies, supporting the use of ultrasound imaging techniques to improve diagnostic accuracy in distinguishing DCIS from IDC, while also highlighting the added value of AI-driven approaches.

Specific sonographic features defined by the ACR BI-RADS lexicon—shape, orientation, and margin characteristics—are reliable indicators for distinguishing between benign and malignant breast masses, confirming their effectiveness in predicting the nature of a breast mass [27,31]. A previous study demonstrated that using a deep learning network for semantic segmentation of breast ultrasound images based on the BI-RADS lexicon achieved high predictive accuracy for IDC and DCIS, with an accuracy of 91.5%, sensitivity of 88.6%, and specificity of 91.8% [32]. This approach is particularly valuable for distinguishing DCIS from IDC, as early and accurate detection of these conditions is critical for guiding treatment decisions [32]. Although ACR BI-RADS ultrasound descriptors alone demonstrated modest performance, combining them with LND and lesion area in a multiparametric model substantially improved discrimination between mass-type DCIS and IDC.

Lesion size plays a crucial role in differentiating DCIS from more invasive stages, making it a critical factor in breast cancer diagnosis [33,34]. Jong Won Lee et al. identified preoperative factors linked to the upstaging of DCIS to invasive cancer, emphasizing that a sonographic lesion size > 2 cm significantly increases the risk and may indicate the need for sentinel lymph node biopsy [33]. Similarly, another study confirmed that lesion size is a critical risk factor for the upstaging of breast DCIS, with lesions larger than 2 cm demonstrating a higher likelihood of invasiveness [34]. In breast cancer, using area (as opposed to just length) provides a more accurate representation of tumor size, offering a more comprehensive understanding of the lesion’s two-dimensional dimensions. Our findings align with previous studies, validating that larger breast lesions are strongly associated with IDC.

Differentiating DCIS from IDC remains a significant challenge, even for experienced specialists. However, the integration of AI has significantly enhanced the accuracy of breast ultrasound diagnostics for DCIS, while also aiding in the differentiation of other breast diseases [8,34]. A generative AI model demonstrated substantial improvements, increasing DCIS specificity and sensitivity by 43.0% and 16.5%, respectively, compared to radiologist interpretations [34]. A deep learning model outperformed radiologists in predicting DCIS, achieving greater diagnostic accuracy with an AUC of 0.802 [8]. These findings underscore the transformative potential of AI-driven approaches in advancing breast ultrasound diagnostics for DCIS.

Numerous studies have demonstrated the value of multi-parametric approaches in improving DCIS diagnosis [34,35]. Qinghua Niu et al. developed a predictive nomogram (AUC 0.889) incorporating ultrasonographic features such as lesion morphology, stiffness, vascularity, and perfusion [34]. Another study demonstrated that nomograms integrating B-mode ultrasound and CEUS features effectively detected DCIS microinvasion preoperatively, achieving AUCs of 0.850, 0.848, and 0.879 in training and validation sets [35]. Our comprehensive LND nomogram achieved comparable predictive performance with AUCs of 0.851 for the training set and 0.842 for the validation set, suggesting improved discriminative performance when integrating clinical measurements with AI-derived features. These findings emphasize the importance of integrating AI-generated ultrasound lexicon with clinical parameters to achieve optimal diagnostic accuracy in IDC and DCIS.

Initially developed using AI-assisted imaging analysis, the LND nomogram may help in the pre-biopsy differentiation of mass-type DCIS from IDC based on basic ultrasound measurements. Unlike AI-based systems, it does not require specialized software and can be used manually or with simple calculators, making it accessible in settings without advanced technology. By potentially improving diagnostic accuracy, it may inform treatment planning, reduce unnecessary sentinel lymph node biopsies in low-risk cases, and support safer, more cost-effective care.

### Limitation

This study has several limitations. First, the cohort was restricted to mass-type lesions suitable for BI-RADS classification, potentially excluding non-mass patterns often observed in DCIS. Second, analysis was limited to a single AI platform (S-Detect, Samsung) without cross-vendor validation, which may restrict generalizability. Although imaging was conducted under the supervision of sonographers, our previous study demonstrated that S-Detect achieved diagnostic performance comparable to that of expert radiologists, thereby supporting its potential as a reliable and clinically applicable AI-assisted diagnostic tool [20]. The retrospective, single-center design also limits the applicability of findings to broader clinical settings. In addition, the relatively small number of DCIS cases may have reduced statistical power and affected model stability. To enhance model reliability, we applied 1000 bootstrap resamples for internal validation, a widely accepted approach for small datasets. Future multi-center external validation with larger datasets is needed to confirm the generalizability and robustness of our findings across diverse patient populations and clinical settings.

The study did not compare the nomogram with deep learning models, which should be explored in future work. Moreover, advanced evaluation metrics—such as net reclassification improvement (NRI), integrated discrimination improvement (IDI), and decision curve analysis (DCA)—were not included but warrant incorporation in future validation studies. These limitations may be addressed by including non-mass lesion types, validating across multiple AI systems, applying structured quality assurance protocols, and conducting prospective multicenter studies with larger cohorts to enhance robustness and generalizability.

## 5. Conclusions

The AI-based ultrasound nomogram showed reliable performance in distinguishing mass-type DCIS from invasive ductal carcinoma. However, given the retrospective study design, multi-center validation using various databases is essential to avoid bias and establish clinical utility.

## Figures and Tables

**Table 1:** Baseline characteristics of training and validation cohorts.

Characteristics	Training (*n* = 122)	Validation (*n* = 53)	*p*-Value
**Mean patient age ± SD**	53.2 ± 13.5 *	54.7 ± 12.8 *	0.470
Age group			0.900
≤50	35 (28.7)	14 (26.4)	
>50	87 (71.3)	39 (73.6)	
Pathology result			0.863
DCIS	19 (15.6)	7 (13.2)	
IDC	103 (84.4)	46 (86.8)	
LND (cm)			0.760
≤3	76 (62.3)	31 (58.5)	
>3	46 (37.7)	22 (41.5)	
**Area (cm^2^) mean ± SD**	2.33 ± 2.38 *	2.32 ± 2.07 *	0.416
**Depth (cm) mean ± SD**	0.65 ± 0.36 *	0.71 ± 0.37 *	0.890
Shape			0.776
Oval	16 (13.1)	5 (9.4)	
Round	9 (7.4)	4 (7.5)	
Lobular	5 (4.1)	1 (1.9)	
Irregular	92 (75.4)	43 (81.1)	
Orientation			0.784
Non-Parallel	10 (8.2)	3 (5.7)	
Parallel	112 (91.8)	50 (94.3)	
Margin			0.730
Circumscribed	51 (41.8)	24 (45.3)	
Indistinct	17 (13.9)	9 (17.0)	
Angular	16 (13.1)	8 (15.1)	
Microlobulated	30 (24.6)	8 (15.1)	
Spiculated	8 (6.6)	4 (7.5)	
Posterior Features			0.057
Shadowing	33 (27.1)	8 (15.1)	
Enhancement	83 (68.0)	38 (71.7)	
Combined ^†^	6 (4.9)	7 (13.2)	
Echo pattern			0.778
Hyperechoic	2 (1.6)	1 (1.9)	
Isoechoic	18 (14.8)	5 (9.4)	
Hypoechoic	99 (81.1)	45 (84.9)	
Complex	3 (2.5)	2 (3.8)	

Note: Unless otherwise specified, data are numbers of lesions, with percentages in parentheses. DCIS = ductal carcinoma in situ, IDC = invasive ductal carcinoma. * Data are means ± standard deviations. ^†^ Combined pattern includes both shadowing and enhancement.

**Table 2:** Univariate and multivariate logistic regression analysis of factors predicting ductal carcinoma in situ in the training set (*n* = 122).

Variables	Univariable Analysis			Multivariable Analysis		
	OR	95% CI	*p*-Value	OR	95% CI	*p*-Value
**Age group**						
**≤50**	2.665	(0.976, 7.277)	0.056	2.063	(0.650, 6.547)	0.219
**>50**	1			1		
**Lesion-to-nipple distance (cm)**						
**≤3**	6.339	(1.392, 28.876)	0.017 *	9.244	(1.778, 48.087)	0.011 *
**>3**	1			1		
**Area (cm^2^)**	0.279	(0.092, 0.847)	0.024 *	0.213	(0.066, 0.686)	0.012 *
**Depth (cm)**	1.187	(0.612, 2.299)	0.612			
**Shape**						
**Irregular**	7.052	(0.901, 55.290)	0.063 *	9.736	(1.088, 87.965)	0.042 *
**Other ^†^**	1			1		
**Orientation**						
**Parallel**	1					
**Non-Parallel**	1.723	(0.205, 14.466)	0.616			
**Margin**						
**Circumscribed**	3.125	(0.813, 12.017)	0.097			
**Indistinct**	1.875	(0.545, 6.449)	0.319			
**Microlobulated**	1					
**Angular/Spiculated**	0.682	(0.127, 12.017)	0.655			
**Posterior Features**						
**Shadowing**	1					
**No Shadowing** **^‡^**	1.470	(0.208, 2.222)	0.524			
**Echo pattern**						
**Hypoechoic**	1			1		
**Others** **^&^**	3.172	(1.084, 9.282)	0.035 *	3.427	(0.984, 11.934)	0.066

Note: OR = odds ratio, CI = confidence interval. Variables with *p* < 0.10 in univariable analysis were included in the multivariable model. ^†^ Other shapes include oval, round, and lobular. ^‡^ Includes enhancement and combined patterns. ^&^ Other echo patterns include hyperechoic, isoechoic, and complex cystic and solid. * *p* < 0.05.

## Data Availability

The data presented in this study are not publicly available due to confidentiality and ethical issues. They are available upon request from the corresponding author.

## Supplementary Materials

The following supporting information can be downloaded at: https://www.mdpi.com/article/10.3390/cancers17152497/s1, Figure S1: Model calibration assessment based on mean absolute error (MAE) between predicted and observed probabilities of DCIS. The graph shows calibration performance for the training (red, *n* = 122) and validation (green, *n* = 53) cohorts across predicted probability intervals (0.0–0.8), evaluated using 1000 bootstrap resamples. Both MAE curves remain below the 0.05 threshold (blue dashed line), indicating strong agreement between predicted and actual DCIS probabilities. Notably, the model exhibits especially reliable calibration in the 0.1–0.5 range—considered the most clinically actionable risk zone—supporting its potential utility for individualized preoperative decision-making (MAE: training = 0.016; validation = 0.034).

## Abbreviations

The following abbreviations are used in this manuscript:

AI	Artificial Intelligence
AUC	Area Under the Curve
CI	Confidence Interval
DCIS	Ductal Carcinoma In Situ
MAE	Mean Absolute Error
OR	Odds Ratio
ROC	Receiver Operating Characteristic
SD	Standard Deviation
