# Supplementary material for: AI-Based Ultrasound Nomogram for Differentiating Invasive from Non-Invasive Breast Cancer Masses
Source: Cancers (Basel). 2025 Jul 29;17(15):2497. doi: 10.3390/cancers17152497 (PMC12345751; doi:10.3390/cancers17152497)
Supplement: Supplementary file 1 [file cancers-17-02497-s001.zip › cancers-3705676-supplementary.pdf]

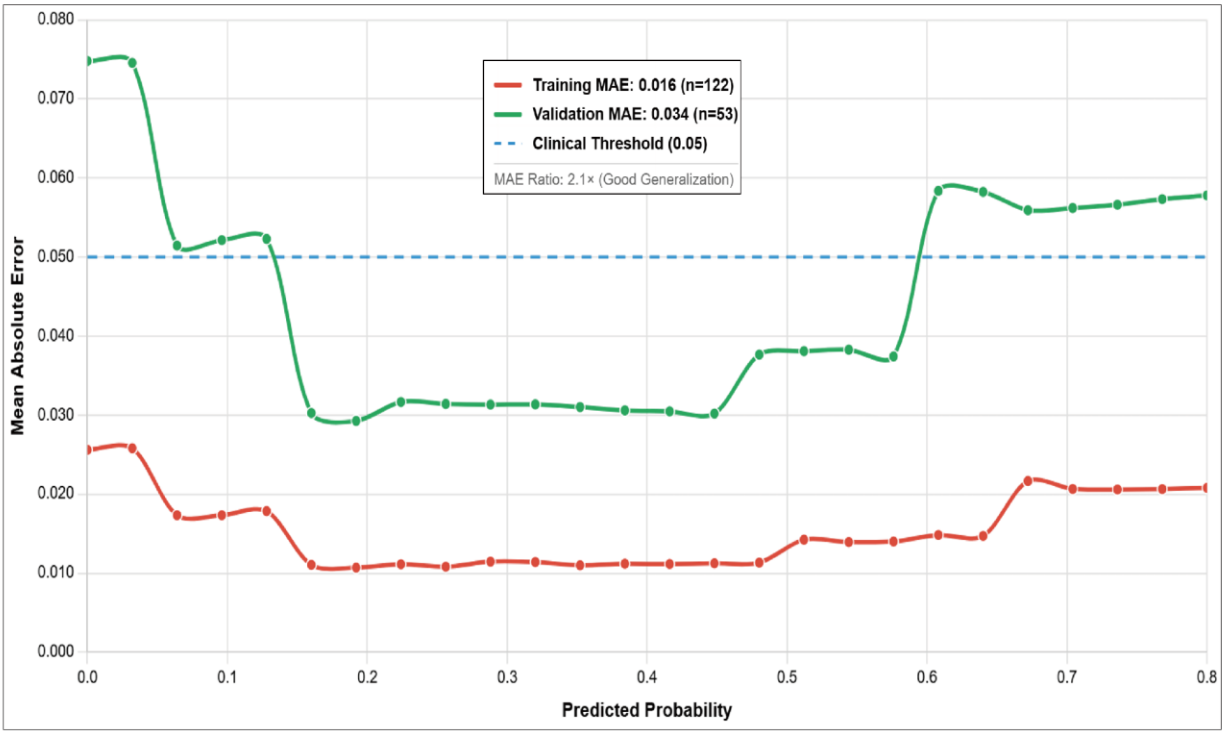

Figure S1. Model calibration assessment based on mean absolute error (MAE) between predicted and observed probabilities of DCIS. The graph shows calibration performance for the training (red, n=122) and validation (green, n=53) cohorts across predicted probability intervals (0.0–0.8), evaluated using 1,000 bootstrap resamples. Both MAE curves remain below the 0.05 threshold (blue dashed line), indicating strong agreement between predicted and actual DCIS probabilities. Notably, the model exhibits especially reliable calibration in the 0.1–0.5 range—considered the most clinically actionable risk zone—supporting its potential utility for individualized preoperative decision-making (MAE: training = 0.016; validation = 0.034).
